# Non‐invasive omics analysis delineates molecular changes in water‐only fasting and its sex‐discriminating features in metabolic syndrome patients

**DOI:** 10.1002/mco2.393

**Published:** 2023-11-02

**Authors:** Yanyu Jiang, Zhimei Tang, Xiaogang Zhu, Biying Xiao, Hechuan Tian, Xingxing Lei, Huan Peng, Jun Qin, Yanmei Zhang, Robert M. Hoffman, Xiaorong Hu, Qiu Chen, Guang Ji, Lijun Jia

**Affiliations:** ^1^ Cancer Institute Longhua Hospital Shanghai University of Traditional Chinese Medicine Shanghai China; ^2^ Hospital of Chengdu University of Traditional Chinese Medicine Sichuan China; ^3^ Dujiangyan Diabetes Hospital Sichuan China; ^4^ Dujiangyan Diabetes Rongxin Hospital of Traditional Chinese Medicine Sichuan China; ^5^ State Key Laboratory of Proteomics Beijing Proteome Research Center National Center for Protein Sciences (Beijing) Beijing Institute of Lifeomics Beijing China; ^6^ Department of Laboratory Medicine Huadong Hospital Fudan University Shanghai China; ^7^ Department of Surgery University of California San Diego San Diego California USA; ^8^ AntiCancer Inc. San Diego California USA; ^9^ Institute of Digestive Diseases Longhua Hospital Shanghai University of Traditional Chinese Medicine Shanghai China

**Keywords:** CD14, integrative omics analyses, metabolic profiling, metabolic syndrome, sex‐discriminating molecular feature, water‐only fasting, weight loss

## Abstract

Fasting has been grown in popularity with multiple potential benefits. However, very few studies dynamically monitor physiological and pathological changes during long‐term fasting using noninvasive methods. In the present study, we recruited 37 individuals with metabolic syndrome to engage in a 5‐day water‐only fasting regimen, and simultaneously captured the molecular alterations through urinary proteomics and metabolomics. Our findings reveal that water‐only fasting significantly mitigated metabolic syndrome‐related risk markers, such as body weight, body mass index, abdominal circumference, blood pressure, and fasting blood glucose levels in metabolic syndrome patients. Indicators of liver and renal function remained within the normal range, with the exception of uric acid. Notably, inflammatory response was inhibited during the water‐only fasting period, as evidenced by a decrease in the human monocyte differentiation antigen CD14. Intriguingly, glycolysis, tricarboxylic acid cycle, and oxidative phosphorylation underwent a sex‐dependent reprogramming throughout the fasting period, whereby males exhibited a greater upregulation of carbohydrate metabolism‐related enzymes than females. This disparity may be attributed to evolutionary pressures. Collectively, our study sheds light on the beneficial physiological effects and novel dynamic molecular features associated with fasting in individuals with metabolic syndrome using noninvasive methods.

## Graphical abstract

We recruited 37 individuals with metabolic syndrome to engage in a 5‐day water‐only fasting regimen, and simultaneously captured the molecular alterations through urinary proteomics and metabolomics. The results showed that the inflammatory response was inhibited during the water‐only fasting period. Interestingly, glycolysis, tricarboxylic acid cycle, and oxidative phosphorylation underwent a sex‐dependent reprogramming throughout the fasting period, whereby males exhibited a greater upregulation of carbohydrate metabolism‐related enzymes than females.

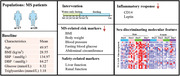

## 1 INTRODUCTION

Obesity, which refers to an imbalance between body weight and height, is currently recognized as a global pandemic.^1^, ^2^, ^3^ Obesity is often associated with the development of hyperlipidemia, diabetes, hypertension, insulin resistance, and other pathological symptoms.^1^, ^2^, ^3^ Some of these comorbidities are hallmarks of the metabolic syndrome (MS).^1^ Presently, the prevalence of obesity or MS ranges from 10% to 40% throughout the world.^4^ The surge in obesity and MS cases can be attributed to the adoption of high‐glucose and high‐fat diets, coupled with reduced physical activity.^5^ Excessive consumption of glucose and fat leads to systemic changes, including elevated levels of glucose, lipids, and inflammatory factors, as well as the onset of insulin resistance.^4^, ^6^ Consequently, the likelihood of developing fatty liver disease, cardiovascular disease, and cancer is significantly heightened.^4^, ^6^ Multiple approaches and treatments have been employed for weight loss, encompassing dietary control, physical activity, pharmacotherapy, bariatric surgery, and microbial‐based therapies.^1^, ^7^, ^8^ However, pharmacotherapy and bariatric surgery are accompanied by unwanted adverse effects and some risks. Therefore, the most widely accepted nonpharmacological intervention remains dietary control and exercise.

Accumulated studies have consistently demonstrated the effectiveness of intermittent fasting (IF, including alternate day fasting and the 5:2 diet), as well as calorie restriction (CR) (restricting intake to 60%−80% of energy needs daily) for weight loss. The average weight loss ranges from 0.2 to 0.5 kg per week in individuals with overweight or obese (body mass index, BMI > 25 kg/m^2^).^9^, ^10^, ^11^, ^12^ Some studies suggest that IF may result in greater weight loss compared to CR alone.^13^, ^14^ Time‐restricted eating, which involves limiting meal timing to a window of 4−10 h per day, appears to yield lower weight loss (approximately 3%−4% reduction from baseline) compared to IF (about 4%−8% reduction from baseline).^9^, ^15^ Although IF or CR has been recommended as an effective method for weight loss, these two methods require participants to take much longer time periods to accomplish. And long‐term fasting (water‐only fasting for more than 3 days) remains infrequent among obesity or MS patients. In our previous study, we investigated the effects of 5‐day water‐only fasting on body weight in normal‐weight individuals. We observed a significant reduction in body weight by 6.8%, and this effect persisted for up to 1 month, with a 2.2% weight loss after 1 month of refeeding.^16^ However, it is important to note that the weight loss effects of fasting differ among individuals with different weight statuses. For instance, IF has been found to produce 0.2−0.5 kg of weight loss per week in overweight or obese individuals (BMI > 25 kg/m^2^),^9^, ^10^, ^11^, ^12^ while it only facilitates approximately 0.2 kg of weight loss per week in normal‐weight individuals (BMI 18.5−24.9 kg/m^2^).^9^, ^17^ Furthermore, patients with obesity or MS are different from normal‐weight individuals, who are healthy without any accumulated disorders. Consequently, the weight‐loss effects and safety of 5‐day water‐only fasting in obese and MS patients still need to be clarified.

Continuous monitoring of physiological and pathological changes is crucial throughout long‐term fasting trials. However, repeated blood collection is often impractical and poorly tolerated by most individuals. Urine samples offer several advantages over blood samples. First, urine collection is noninvasive and is generally accepted by nearly all individuals. Second, large volumes of urine can be obtained without much difficulty. Third, urinary protein levels exhibit a relatively narrower dynamic range. Last, urine proteomic and metabolomic profiling have been used as biochemical tools to identify reliable urinary biomarkers and predict therapy response in human diseases.^18^, ^19^, ^20^

In the present study, we present findings that highlight the benefits, safety, and a novel sex‐related molecular feature of 5‐day water‐only fasting in patients with MS using urine proteomics and metabolomic profiling, a noninvasive approach to dynamically monitor physiological and pathological changes.

## 2 RESULTS

### 2.1 Water‐only fasting reduced body weight and MS‐related risk markers

**FIGURE 1:**
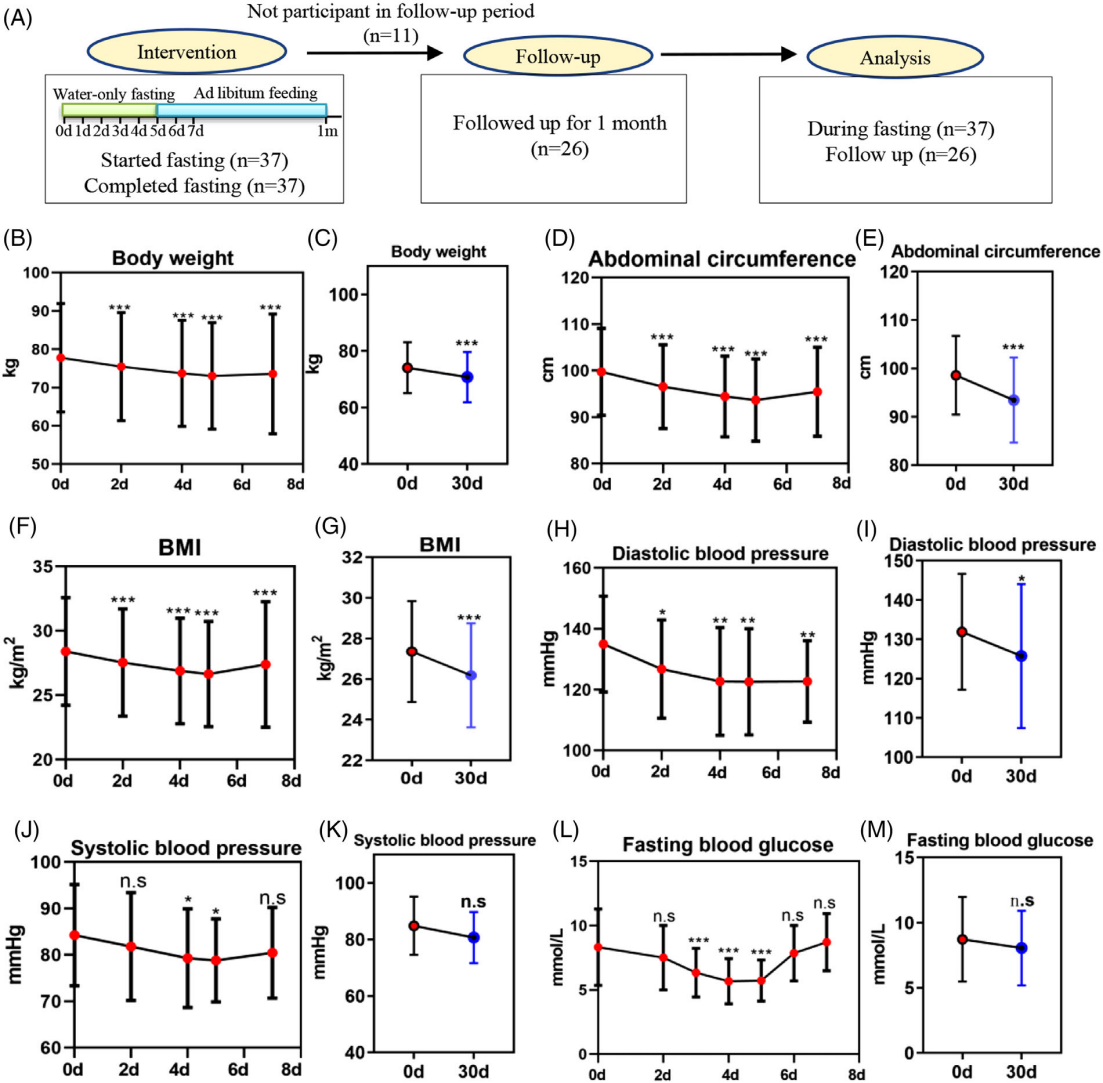
Water‐only fasting reduced body weight and metabolic syndrome‐related risk markers. (A) Protocol of the water‐only fasting study. Thirty‐seven volunteers were eligible based on the inclusion and exclusion criteria. (B–M) Body weight, abdominal circumference, body mass index (BMI), systolic blood pressure, diastolic blood pressure, and fasting blood glucose were measured before, during, and after 5‐day water‐only fasting. n.s denotes not significant, ^*^ *p* < 0.05, ^**^ *p* < 0.01, ^***^ *p* < 0.001.

The study flowchart is presented in Figure 1A, while Table 1 shows the baseline characteristics of the enrolled MS patients. There were 20 male and 17 female participants, all of whom met the inclusion criteria described in the materials and methods.

**TABLE 1:** Baseline characteristics of the enrolled metabolic syndrome patients (*n* = 37).

Characteristics	Mean ± SD
Gender	Male: 20; female: 17
Age	49.97 ± 10.50
Weight (kg)	77.83 ± 14.24
Waist circumference (cm)	99.77 ± 9.34
BMI (kg/m^2^)	28.55 ± 4.22
SBP (mmHg)	134.97 ± 15.81
DBP (mmHg)	84.27 ± 10.90
Glucose (mmol/L)	8.32 ± 2.97
Cholesterol (mmol/L)	4.43 ± 0.78
Triglycerides (mmol/L)	3.18 ± 2.69
HDL (mmol/L)	1.87 ± 0.53
LDL (mmol/L)	2.35 ± 0.63
Fatty liver	Yes: 28

Abbreviations: BMI, body mass index; DBP, diastolic blood pressure; HDL, high‐density lipoprotein; LDL, low‐density lipoprotein; SBP, systolic blood pressure; SD, standard deviation.

MS‐related risk markers were measured before and after the 5‐day water‐only fasting period. Following the fast, significant reductions were observed in body weight (4.23 kg, *p* < 0.001), abdominal circumference (7.24 cm, *p* < 0.001), and BMI (2.60 kg/m^2^, *p* < 0.001) (Figure 1B,D,F). Even after refeeding for 1 month, these three indexes remained lower than the baseline levels (Figure 1C,E,G). In addition, there was a significant decrease in diastolic blood pressure (DBP, 9.81 mmHg, *p* < 0.001) and systolic blood pressure (SBP, 2.42 mmHg, *p* = 0.013) during water‐only fasting (Figure 1H,J). After refeeding for 1 month, DBP remained lower than the baseline levels (Figure 1I), while SBP recovered back to the baseline levels (Figure 1K). Furthermore, fasting blood glucose significantly decreased by 2.60 mmol/L after a 5‐day water‐only fast (*p* < 0.001) (Figure 1L,M). All fasting blood glucose remained above 3.3 mmol/L during the water‐only fasting, without hypoglycemia occurring (Figure 1L,M). These data collectively indicate that water‐only fasting rapidly reduced body weight and MS‐related risk markers.

### 2.2 Safety‐associated indexes during water‐only fasting

Table 2 presents the safety‐associated indexes during the 5‐day water‐only fasting period. First, in terms of liver function, albumin, and the albumin/globulin ratio increased compared to the baseline level after the fasting period. After 1 month of refeeding, albumin returned to baseline levels, while the albumin/globulin ratio remained elevated compared to baseline levels. Other liver‐function‐related indexes remained within the normal range, with a slight decrease in direct bilirubin, total protein, and globulin after 1 month of refeeding.

**TABLE 2:** Safety‐associated indexes during 5‐day water‐only fasting.

	Mean ± SD			*p*‐Value (*t*‐test)		
Index	Day 0	Day 7	Day 30	Day 0 versus day 7	Day 0 versus day 30	Day 7 versus day 30
Liver function						
Total bilirubin (μmol/L)	22.64 ± 19.94	16.86 ± 6.73	16.16 ± 7.84	0.1330	0.0609	0.7327
Direct bilirubin (μmol/L)	10.44 ± 12.20	6.40 ± 2.45	6.43 ± 4.77	0.0784	0.0366	0.9739
Indirect bilirubin (μmol/L)	12.39 ± 9.12	10.46 ± 6.02	9.87 ± 4.72	0.3047	0.1342	0.6948
Total protein (g/L)	74.43 ± 8.19	72.90 ± 5.23	71.12 ± 8.07	0.3869	0.0371	0.3505
Albumin (g/L)	43.43 ± 2.80	44.60 ± 2.78	43.53 ± 3.20	0.0285	0.8649	0.2024
Globulin (g/L)	31.00 ± 7.40	28.30 ± 4.09	27.60 ± 5.83	0.0736	0.0098	0.6167
Albumin/globulin	1.45 ± 0.28	1.61 ± 0.22	1.67 ± 0.51	0.0026	0.0136	0.5758
Alanine aminotransferase (U/L)	23.89 ± 21.66	24.63 ± 22.49	25.20 ± 11.81	0.8088	0.7532	0.9095
Aspartate aminotransferase (U/L)	34.01 ± 30.24	33.55 ± 16.54	29.06 ± 11.02	0.9357	0.4310	0.2543
Alkaline phosphatase (U/L)	107.42 ± 38.08	112.73 ± 28.98	97.46 ± 38.24	0.5483	0.3140	0.1109
Γ‐Glutamyl transpeptidase (U/L)	38.21 ± 24.09	37.42 ± 19.65	36.22 ± 26.55	0.8018	0.4522	0.8538
Renal function						
Urea (mmol/L)	6.75 ± 1.51	5.97 ± 1.30	6.32 ± 1.23	0.0293	0.1744	0.3185
Creatinine (μmol/L)	77.50 ± 10.22	82.00 ± 14.21	77.12 ± 12.19	0.0028	0.7737	0.1893
Uric acid (μmol/L)	303.65 ± 76.49	533.58 ± 135.68	319.73 ± 75.57	<0.0001	0.3956	<0.0001

*Note*: For the comparison, Student's *t*‐test was used. All statistical tests were two‐sided, and *p* ≤ 0.05 was considered statistically significant and is highlighted in red.

Abbreviation: SD, standard deviation.

Second, regarding renal function, urea levels decreased by 0.78 mmol/L, while creatinine levels increased by 4.5 μmol/L after the 5‐day water‐only fasting period. Uric acid levels increased from 303.65 to 533.58 μmol/L during the fasting period and then returned to 319.73 μmol/L after the refeeding period (*p* = 0.3956, day 0 vs. day 30). These findings suggest that uric acid‐related symptoms (e.g., gout) should be closely monitored in patients with MS during water‐only fasting.

Last, no serious adverse events, including nausea, diarrhea, fever, stomach ache, or gout, were observed throughout the study. In conclusion, the water‐only fasting regimen demonstrates significant beneficial effects without obvious toxicity in patients with MS.

### 2.3 Water‐only fasting inhibits inflammatory response in MS patients determined by urine proteomic

**FIGURE 2:**
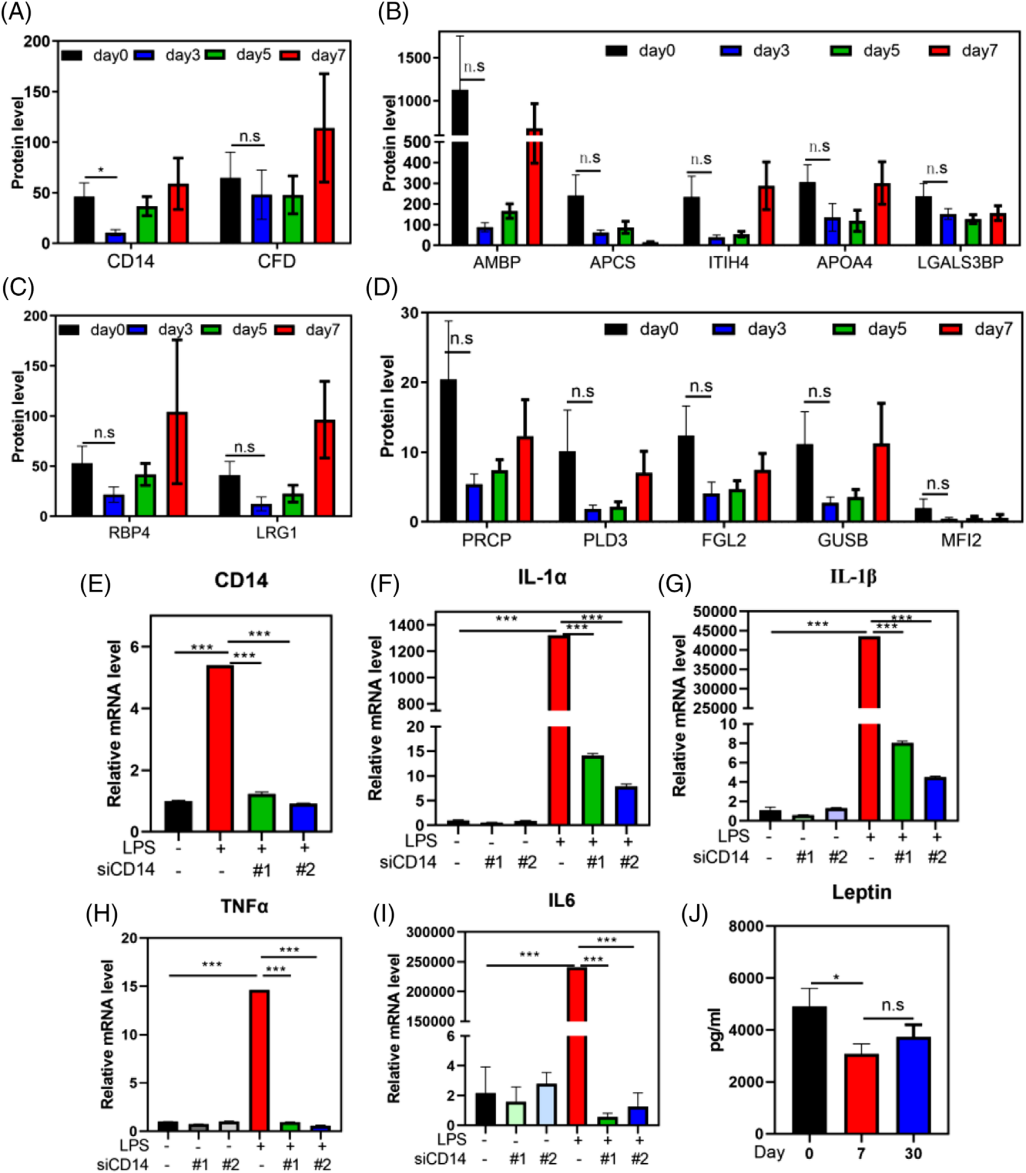
Water‐only fasting inhibits inflammatory response in metabolic syndrome patients at urine proteomic layer. (A–D) Fourteen inflammation‐related urine proteins decreased during 5‐day water‐only fasting. (E–I) Raw264.7 cells were transfected with control or CD14 siRNA and then treated with lipopolysaccharide (LPS; 2 μg/mL) for 6 h. Total RNA was isolated, and qPCR was performed to determine the mRNA level of CD14, interleukin (IL)‐1α, IL‐1β, tumor necrosis factor‐alpha (TNF‐α), and IL‐6. (J) The expression level of leptin was determined using the blood samples. n.s denotes not significant, ^*^ *p* < 0.05, ^***^ *p* < 0.001.

Our previous study demonstrated that water‐only fasting increases the frequency of anti‐inflammatory Treg cells in blood of normal‐weight individuals.^16^ However, the anti‐inflammatory effects of water‐only fasting at the urinary protein level and in patients with MS remain unknown. We identified 16 inflammation‐related urine proteins using noninvasive approaches and observed that 87.50% (14/16) of these proteins decreased during the water‐only fasting period (Figure 2A–D). Among these proteins, human monocyte differentiation antigen CD14 exhibited a significant decrease (*p* < 0.05, Figure 2A). CD14 is a glycolipid‐anchored membrane glycoprotein that forms a complex with lipopolysaccharide (LPS)‐binding protein and MD2 in response to LPS stimulation.^21^ To further investigate the role of CD14 in the inflammatory response, we downregulated CD14 in monocytes using siRNA, mimicking the effect of water‐only fasting, and observed a significant inhibition in the expression of inflammatory factors, including interleukin (IL)‐1α, IL‐1β, tumor necrosis factor‐alpha (TNF‐α), and IL‐6 (Figure 2E–I). Additionally, given that previous studies have shown that leptin upregulates CD14 in Kupffer cells, and promotes nonalcoholic steatohepatitis (NASH) progression,^22^ we examined the expression of leptin after water‐only fasting. We found a significant decrease in its expression (Figure 2J). These results suggest that water‐only fasting may exhibit anti‐inflammatory and anti‐NASH effects through the inhibition of leptin and CD14 expression.

### 2.4 Water‐only fasting activates carbohydrate metabolism‐related enzymes in MS patients

**FIGURE 3:**
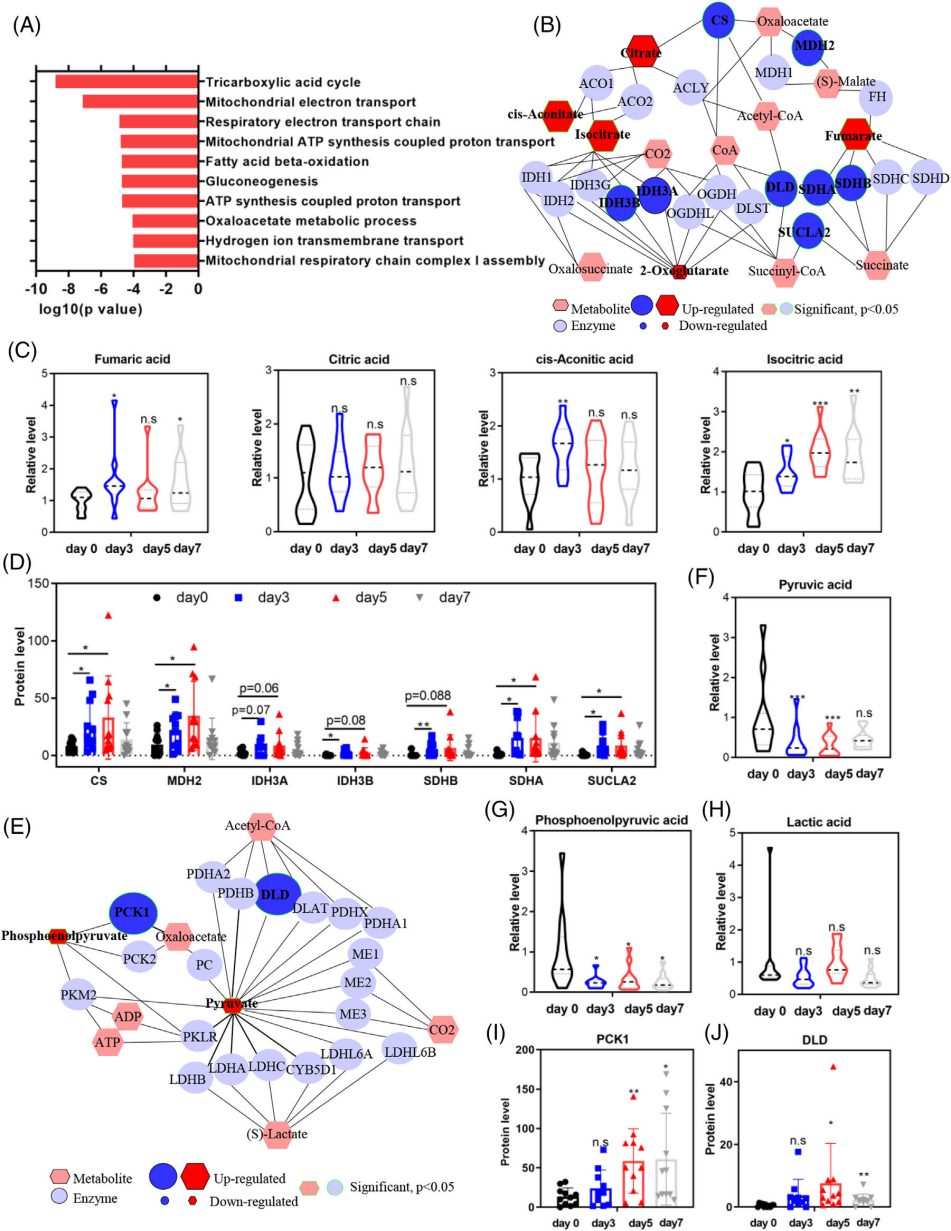
Five‐day water‐only fasting induced metabolic reprogramming. (A) Metabolite set enrichment analysis of different metabolites was performed on the Metaboanalyst website. Differences between multiple groups were analyzed using one‐way analysis of variance (ANOVA). (B and E) Integrated analysis of water‐only fasting was performed using MetScape. (C, D, F–J) Representative metabolites and metabolic enzymes in tricarboxylic acid (TCA) cycle and glycolysis. n.s denotes not significant, ^*^ *p* < 0.05, ^**^ *p* < 0.01, ^***^ *p* < 0.001.

To gain a comprehensive molecular understanding of water‐only fasting in MS patients, consensus component plots generated by the DIABLO algorithm, an integrative multiomics approach,^23^ demonstrated distinct features in urine metabolomic and proteomic profiles between the 3‐ and 5‐day fasting periods (Figure S1). By applying a significance threshold of *p*‐value rate ≤0.05 and mean intensity‐based fraction of total (iFOT) expression ≥0.1, we identified 133 proteins that were significantly upregulated after a water‐only fast. These upregulated proteins predominantly functioned in metabolic pathways, such as the tricarboxylic acid (TCA) cycle, mitochondrial electron transport, fatty acid metabolism, and glucogenesis (Figure 3A).

Integrated analysis of metabolic and proteomic profiles using MetScape revealed the activation of the TCA cycle during water‐only fasting (Figure 3B). Specifically, isocitric acid, cis‐aconitic acid, and fumaric acid were upregulated during water‐only fasting, with only isocitric acid showing sustained elevation beyond the baseline level even after 2 days of refeeding (Figure 3C). The metabolic enzymes involved in the TCA cycle, such as CS, MDH2, IDH3B, SDHA and SDHB, and SUCLA2, were significantly upregulated during the 5‐day water‐only fasting period (Figure 3D). Notably, CS, MDH2, SDHA, and SUCLA2 continued to exhibit increased expression throughout the water‐only fasting period (Figure 3D).

In the glycolysis pathway, both pyruvate and phosphoenolpyruvate experienced significant decreases, with pyruvate displaying a consistent decline during water‐only fasting (Figure 3E–G). Lactic acid, however, did not demonstrate significant changes (Figure 3H). Interestingly, the metabolic enzymes involved in glycolysis, including dihydrolipoamide dehydrogenase (DLD) and phosphoenolpyruvate carboxykinase (PCK1), significantly increased during water‐only fasting (Figure 3E,I,J). These two enzymes displayed a persistent increase and remained higher than the baseline levels even after 2 days of refeeding (Figure 3I,J). These results collectively indicate the activation of the TCA cycle at the urine metabolic and proteomic levels during fasting, whereas only the metabolic enzymes in glycolysis exhibit activation.

### 2.5 Sex‐discriminating molecular features in MS patients during water‐only fasting

**FIGURE 4:**
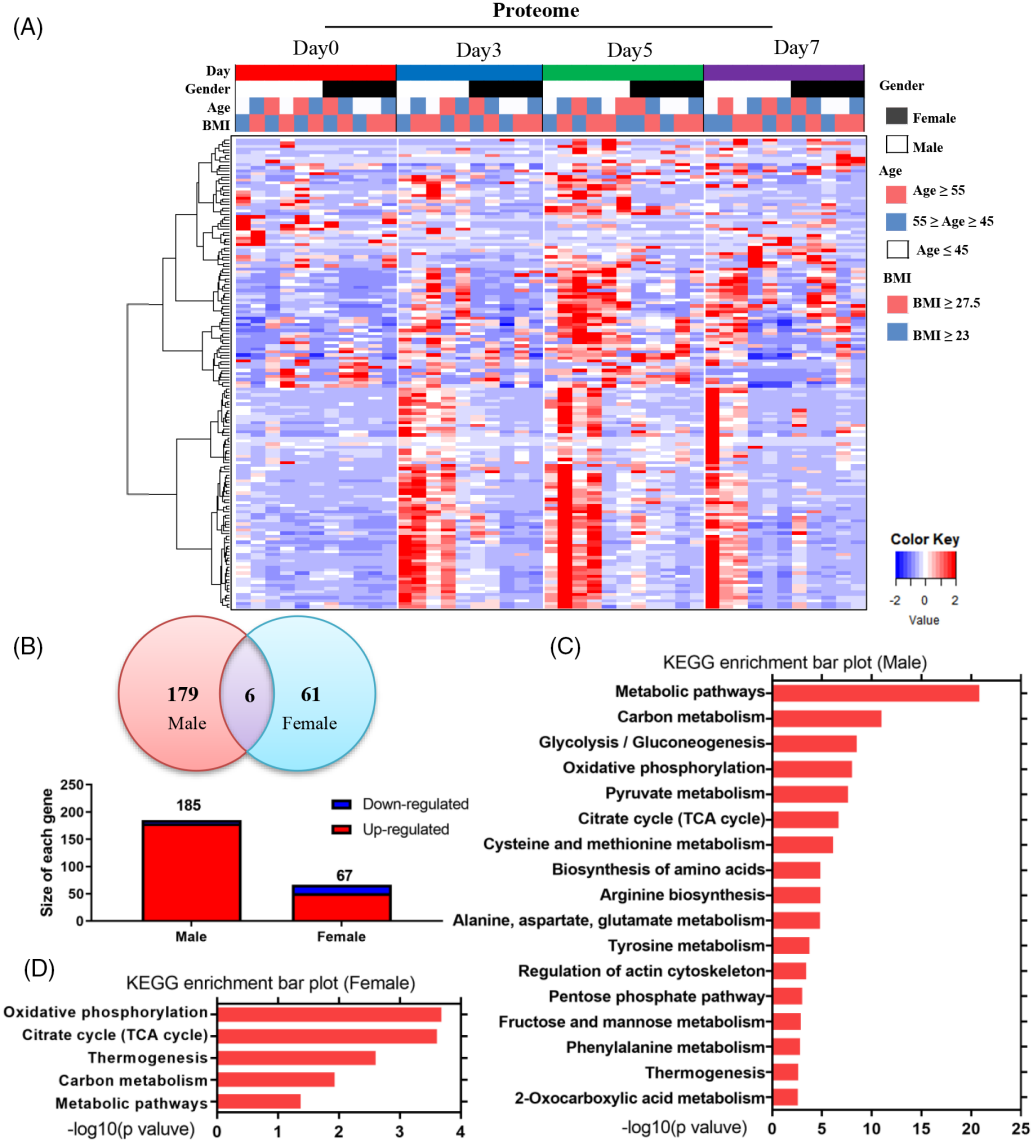
The sex‐discriminating molecular features in metabolic syndrome patients during water‐only fasting. (A) Heatmap analysis of all significantly differentially altered urinary proteins, including day 0 (*n* = 11), day 3 (*n* = 10), day 5 (*n* = 11), and day 7 (*n* = 11). (B) Venn analysis of differently altered urinary proteins in males and females (day 0 vs. day 5, *p* < 0.05). (C and D) KEGG analysis of the differentially regulated proteins in males and females. BMI, body mass index; TCA, tricarboxylic acid.

During heatmap analysis, we made an intriguing observation that the altered urinary proteins during the 5‐day water‐only fasting period were closely associated with sex rather than age or BMI (Figure 4A). This suggests the presence of novel sex‐discriminating molecular features in response to water‐only fasting. Venn analysis further revealed that 185 proteins (179 upregulated and six downregulated) were altered in males, while only 67 proteins (51 upregulated and 16 downregulated) showed changes in females (Figure 4B). Kyoto Encyclopedia of Genes and Genomes (KEGG) analysis in males demonstrated that differentially‐regulated proteins predominantly clustered in metabolic pathways, including glycolysis, oxidative phosphorylation, pyruvate metabolism, and the TCA cycle (Figure 4C). Conversely, the differentially‐regulated proteins in females primarily clustered in oxidative phosphorylation, the TCA cycle, thermogenesis, and carbon metabolism (Figure 4D).

**FIGURE 5:**
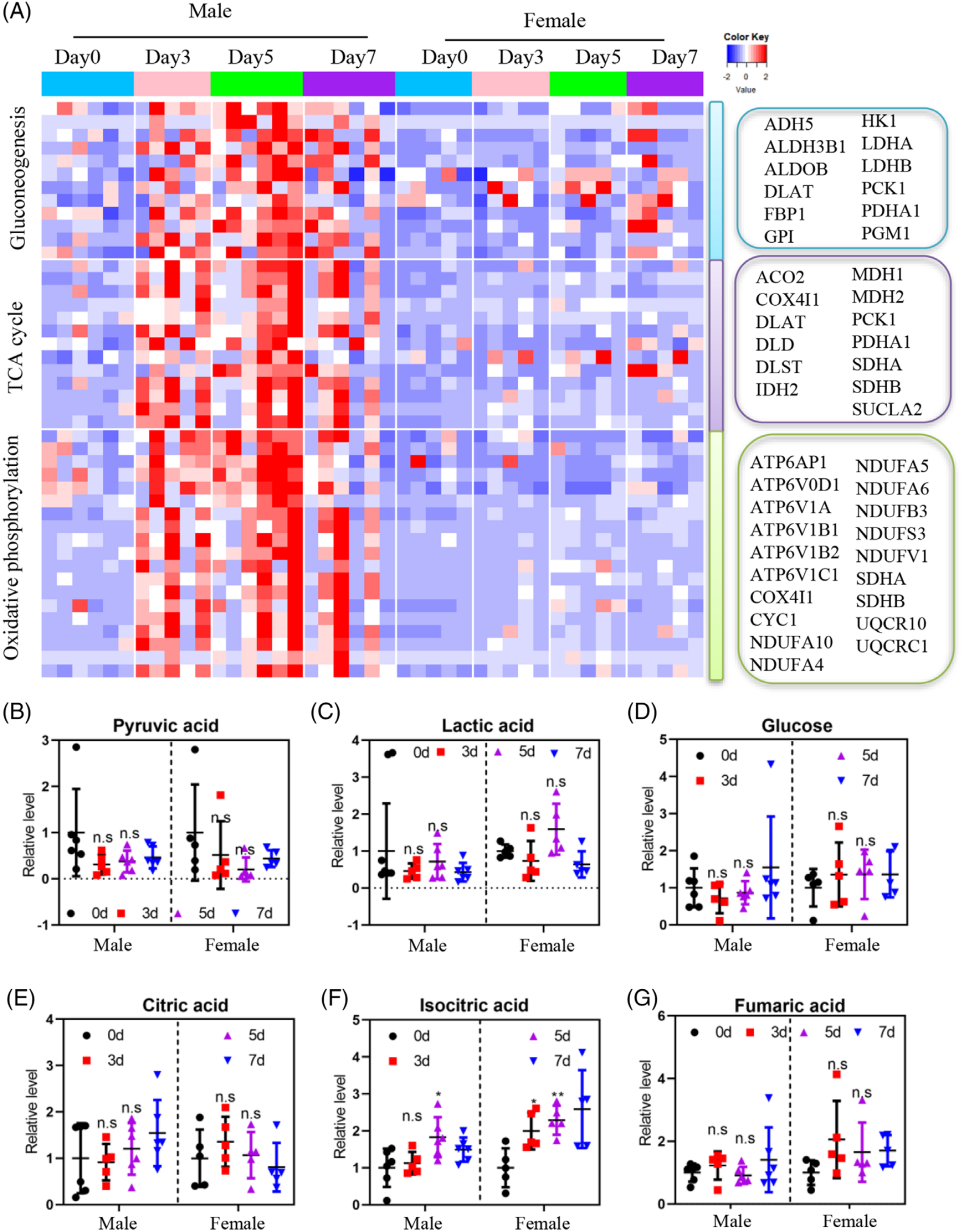
Males showed a greater upregulation of metabolic enzymes related to carbohydrate metabolism compared to females during the 5‐day water‐only fasting. (A) Heatmap analysis of differentially regulated proteins in glucose metabolic pathways between males and females. (B–G) Representative metabolites during water‐only fasting in both males and females. TCA, tricarboxylic acid. n.s denotes not significant, ^*^ *p* < 0.05, ^**^ *p* < 0.01.

To gain a more detailed understanding of the sex‐related differences, we conducted quantitative analysis focusing on key metabolic pathways such as glycolysis, oxidative phosphorylation, and the TCA cycle in males and females. Consistent with the sex difference, the heatmap of glucose metabolism‐related enzymes displayed upregulation in males, with nearly all enzymes showing a sustained increase throughout the water‐only fasting period. Even after 2 days of refeeding, the enzyme levels remained higher than the baseline (Figure 5A). In contrast, only a few glucose‐related enzymes were upregulated in females (Figure 5A). These findings further support the presence of sex‐discriminating molecular features in MS patients during water‐only fasting. However, metabolites such as pyruvic acid, lactic acid, glucose, citric acid, isocitric acid, and fumaric acid did not show significant differences between males and females (Figure 5B–G). These results highlight the existence of sex‐biased differences in the utilization of carbohydrate as energy sources during water‐only fasting in MS patients determined by urine proteomic.

## 3 DISCUSSION

Our previous study demonstrated that a 5‐day water‐only fasting intervention led to a significant reduction in body weight by 6.8% compared to the baseline level in individuals with normal weight.^16^ However, the weight‐loss effects of fasting vary among individuals with different weight statuses. Specifically, IF only resulted in approximately 0.2 kg of weight loss per week in normal‐weight individuals (BMI 18.5–24.9 kg/m^2^),^9^, ^17^ whereas in overweight or obese individuals, it induced approximately 0.2–0.5 kg of weight loss per week.^9^, ^10^, ^11^, ^12^, ^17^ In the present study, we observed an obvious decrease in body weight by 6.12% compared to the baseline level in overweight individuals (mean BMI 28.55 kg/m^2^), which exhibited a similar weight loss effect to that observed in normal‐weight individuals (mean BMI 24.11 kg/^2^).^16^

In addition to weight loss, fasting offers several other benefits, such as reducing MS‐related indexes, as well as inflammation and oxidative stress.^9^, ^10^, ^24^, ^25^, ^26^ However, the effects of fasting on these risk factors vary among studies. For instance, some studies have reported a decrease in both SBP and DBP during fasting,^10^, ^26^ while others have shown no significant effects.^12^ In the present study, we found that a 5‐day water‐only fasting regimen reduced both SBP and DBP in MS patients, which is consistent with the findings in normal‐weight individuals.^16^ Importantly, the 5‐day water‐only fasting approach proved to be effective and safe in MS patients, as demonstrated by the following observations: (1) water‐only fasting mitigated MS‐related risk factors, including body weight, BMI, waistline, SBP, DBP, and blood glucose. Even after 1 month of refeeding, some of these indexes remained lower than the baseline level in the present study. These benefits are comparable to those observed with CR,^27^, ^28^ IF,^12^, ^29^, ^30^ ketogenic diet,^31^, ^32^ and fasting mimicking diet.^33^ (2) Liver and renal functions were maintained within normal ranges after refeeding in MS patients who had already accumulated some disorders, with the exception of uric acid. (3) All fasting blood glucose measures remained above 3.3 mmol/L without any occurrence of hypoglycemia throughout the water‐only fasting period.

Accumulating evidence suggests that CR or IF reduces the levels of circulating pro‐inflammatory cytokines, such as TNF‐α, IL‐6, and IL‐1β.^24^, ^34^ Jordan et al. demonstrated that IF can regulate monocyte numbers by suppressing CCL2 production, thereby improving inflammatory diseases without compromising antimicrobial immunity.^35^ In our previous study, we reported that water‐only fasting upregulated anti‐inflammatory T‐reg cells in normal‐weight individuals.^16^ However, these anti‐inflammatory effects of water‐only fasting are typically assessed using invasive methods, such as blood sampling. Repeated blood collection is often impractical and poorly tolerated by most individuals. In the present study, we employed urinary proteomics profiling, a noninvasive approach to dynamically monitor the physiological and pathological changes in the human body during water‐only fasting. We observed that water‐only fasting suppressed inflammatory responses and reduced inflammation‐related proteins in MS patients. Notably, CD14, an essential factor for LPS‐stimulated inflammatory response was significantly inhibited. Previous studies have reported that leptin can upregulate CD14 and promote the progression of NASH by enhancing responsiveness to LPS.^22^ In the present study, we found that water‐only fasting significantly decreased leptin levels and body weight. Therefore, we hypothesize that water‐only fasting may play a crucial role in NASH by reducing leptin‐mediated CD14 expression and promoting weight loss.

**FIGURE 6:**
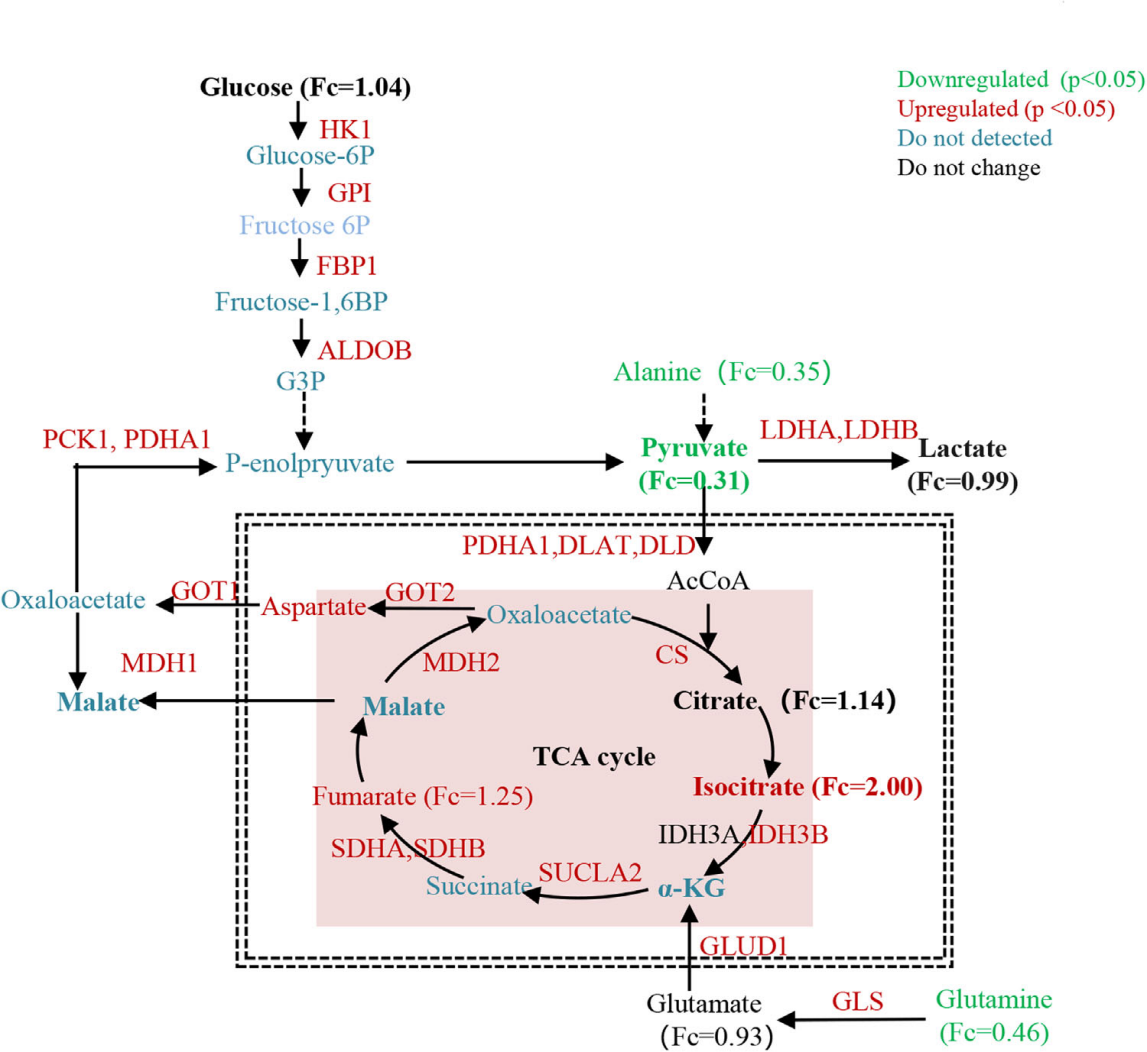
Metabolic response of water‐only fasting in metabolic syndrome patients. TCA, tricarboxylic acid.

In our previous study, we reported that water‐only fasting inhibited glycolysis with a decrease in glucose, pyruvate, and lactate, while the TCA cycle remained activated during the 5‐day water‐only fasting at the blood level.^16^ In the present study, we observed similar metabolic responses, and additionally found that both the TCA cycle and glycolysis‐related enzymes were activated as determined by proteomic profiling during water‐only fasting in MS patients (Figure 6). Importantly, our urinary proteomic omics profiling revealed a sex‐biased response, with males showing a greater upregulation of metabolic enzymes related to carbohydrate metabolism than females during the 5‐day water‐only fasting. Generally, females tend to preserve their energy stores to maintain reproductive capacities in the face of limited food supplies.^36^ In contrast, males utilize their energy reserves for muscle activity, such as hunting or protection.^36^ Therefore, the sex‐discriminating energy partition strategy observed during fasting may be the result of evolutionary pressures, with females prioritizing energy stores essential for reproductive capacities compared to males.^36^

The present study has several limitations that warrant further development. First, the number of participants in the present clinical trial was relatively small. Therefore, a large‐scale clinical trial focusing on sex‐discriminating molecular features of water‐only fasting is needed. Second, our study primarily focused on the changes during water‐only fasting and did not incorporate long‐term approaches to monitor the quality and quantity of food intake during the refeeding period. Third, the trial was conducted in specialized clinics and involved a meditation course, which may have influenced the conclusions drawn in the present study. Last, the individuals who participated in the trial were already interested in fasting or wished to alleviate their MS‐related symptoms through fasting, which may introduce bias.

Overall, our findings collectively demonstrate that water‐only fasting is an effective and economical treatment for MS patients, with the potential to have a significant positive impact on the global obesity and MS pandemic.

## 4 MATERIALS AND METHODS

### 4.1 Participants

All participants signed informed consent forms. The inclusion criteria included any three of the following symptoms: (1) high‐density lipoprotein cholesterol <0.9 mmol/L for males or 1.1 mmol/L for females; (2) triglycerides >1.7 mmol/L; (3) SBP ≥130 mmHg or DBP ≥85 mmHg; (4) fasting blood glucose between 5.6 and 11.1 mmol/L; and (5) BMI > 25 kg/m^2^.

### 4.2 Study design

The clinical trial of water‐only fasting intervention in MS individuals was performed at ZhongZhi Shijiu Dujiangyan Diabetes Hospital (Chengdu, Sichuan, China). In detail, MS patients underwent a water‐only fast, followed by refeeding and a 1‐month follow‐up period. During the 5‐day water‐only fasting intervention, all individuals abstained from consuming any food, with the exception of water intake. To ensure adherence to the fasting intervention, all MS patients received close monitoring from the clinical team and stayed at ZhongZhi Shijiu Dujiangyan Diabetes Hospital during the fasting period. Subsequently, they were permitted to resume their regular diet and lifestyle practices at home during the refeeding period.

### 4.3 Laboratory tests

Laboratory assessments included liver‐ and renal‐function tests, fasting blood glucose, and urine analysis. All test were performed at ZhongZhi Shijiu Dujiangyan Diabetes Hospital.

### 4.4 Urine metabolomic and proteomic profiling

Urine samples were collected and stored at −80°C without any preservatives. For metabolomic profiling, ultraperformance liquid chromatography coupled to a tandem mass spectrometry system (UPLC‐MS/MS) was used to perform metabolic sequencing (Human Metabolomics Institute, Inc, Shenzhen). For proteomic profiling, nano liquid chromatography tandem mass spectrometry system (LC‐MS/M) was used to determine urine proteins by PinealHealth Company. Sample preparation and derivatization protocols were based on previously published methods.^19^, ^20^

### 4.5 Cell culture and reagents

Raw264.7 cells were cultured in Dulbecco's modified Eagle medium (BasalMedia), which contained 10% fetal bovine serum (Biochrom AG) and 1% penicillin–streptomycin. LPS‐B5 (LPS from *Escherichia coli* 055: B5) was purchased from InvivoGen.

### 4.6 Gene silencing

Two siRNA oligonucleotides of CD14 (siCD14#1: GAACUGCAGCAGUGGCUAATT; siCD14#2: GGCUGAAGCAGGUACCUAATT) were synthesized by GenePharma Company. Then, these two siRNAs and their related negative controls were transfected into Raw264.7 cells using Lipofectamine 2000.

### 4.7 Real‐time quantitative polymerase chain reaction analyses

Total RNA was isolated, and real‐time quantitative polymerase chain reaction (qPCR) was performed to determine the mRNA levels of inflammatory factors. First, the Ultrapure RNA kit was used to isolate RNA from Raw264.7 cells. Then, the PrimerScript reverse transcription reagent kit was used to reverse RNA into cDNA (Vazyme Biotech). Finally, we determined the cycle threshold by qPCR using Power SYBR Green PCR MasterMix kit. The primer sequences are shown in Table S2.

### 4.8 Statistical analysis

Differences between two groups were analyzed by Mann–Whitney *U*‐test or the Student's *t*‐test, whereas differences between multiple groups were analyzed using one‐way analysis of variance. *p* < 0.05 represents statistical significance.

## AUTHOR CONTRIBUTIONS

J.L.J., J.G., C.Q., and H.X.R. conceived the general framework of this study. J.Y.Y., T.Z.M., Z.X.G., X.B.Y., T.H.C., L.X.X., P.H., H.X.R., C.Q., and J.L.J. performed the clinical trials. J.Y.Y. and X.B.Y. performed the in vitro experiments. Q.J., Z.Y.M., R.M.H., and J.G. provided technical or material support. Y.Y.J. and X.B.Y. analyzed the data and wrote and revised the manuscript. R.M.H refined the manuscript. J.L.J. reviewed, revised, and finalized the manuscript. All authors have read and approved the final manuscript.

## CONFLICT OF INTEREST STATEMENT

The authors declare that they have no conflicts of interest.

## ETHICS STATEMENT

The protocol of water‐only fasting intervention in MS individuals was approved by the Ethics Committee of Sichuan Hospital of Integrated Chinese and Western Medicine (2017ky‐05) and registered in the Chinese Clinical Trial Registry (ChiCTR1900028277). Written informed consent was obtained from all participants.

## Supporting information

Supporting Information

## Data Availability

The data are available from the corresponding author upon reasonable request.
