# Supplementary material for: Non‐invasive omics analysis delineates molecular changes in water‐only fasting and its sex‐discriminating features in metabolic syndrome patients
Source: MedComm (2020). 2023 Nov 2;4(6):e393. doi: 10.1002/mco2.393 (PMC10622739; doi:10.1002/mco2.393)
Supplement: Supplementary file 1 — Supporting Information [file MCO2-4-e393-s001.pdf]

Supplementary Information

Non-invasive omics analysis delineates molecular changes of water-only fasting and its sex-discriminating features in metabolic syndrome patients

Yanyu Jiang[1#], Zhimei Tang[2#], Xiaogang Zhu[3,4#], Biying Xiao[1], Hechuan Tian[1], Xingxing Lei[2], Huan Peng[2], Jun Qin[5], Yanmei Zhang[6], Robert M. Hoffman[7,8], Xiaorong Hu[3,4*], Qiu Chen[2*], Guang Ji[9*], Lijun Jia[1*]

[1]Cancer Institute, Longhua Hospital, Shanghai University of Traditional Chinese Medicine, Shanghai, China; [2]Hospital of Chengdu University of Traditional Chinese Medicine, Si Chuan, China; [3]Dujiangyan Diabetes Hospital, Sichuan, China; [4]Dujiangyan Diabetes Rongxin Hospital of Traditional Chinese Medicine, Sichuan, China; [5]State Key Laboratory of Proteomics, Beijing Proteome Research Center, National Center for Protein Sciences (Beijing), Beijing Institute of Lifeomics, Beijing 102206, China; [6]Department of Laboratory Medicine, Huadong Hospital, Fudan University, China; [7]Department of Surgery, University of California San Diego, CA, USA; [8]AntiCancer Inc, San Diego, CA, USA; [9]Institute of Digestive Diseases, Longhua Hospital, Shanghai University of Traditional Chinese Medicine, Shanghai, China; [#]These authors contributed equally to this work.

Supplementary Table 1: Baseline characteristics of MS patients whose urine samples were collected to perform urinary proteomics and metabolomics profiling（n=11）

Characteristics	Mean ±SD
Gender	Male:6; Female:5
Age	47.64±12.44
Weight（kg）	74.68±10.99
Waist circumference(cm)	101.95±7.68
BMI (kg/m^2)	28.31±2.51
SBP（mmHg）	136.36±18.44
DBP（mmHg）	87.27±11.64
Glucose (mmol/L)	9.81±2.95
Cholesterol (mmol/L)	4.51±1.02
Triglycerides (mmol/L)	4.12±2.89
HDL (mmol/L)	1.84±0.63
LDL (mmol/L)	1.90±0.88

Supplementary Table 2: The primer sequences for RT-PCR

Murine	forward	reverse
β-actin	ATGGAGGGGAATACAGCCC	TTCTTTGCAGCTCCTTCGTT
CD14	AATCTACCGACCATGGAGCGTG	CAATTGAAAGCGCTGGACCAA
TNFα	AAATTCGAGTGACAAGCCTGTAG	GAGAACCTGGGAGTAGACAAGGT
IL6	CTGCAAGAGACTTCCATCCAG	AGTGGTATAGACAGGTCTGTTGG
IL1α	TGAAGCTCGTCAGGCAGAAG	CGCTCACGAACAGTTGTGAA
IL1β	TGGCAACTGTTCCTGAACTC	GAAGCTGGATGCTCTCATCA

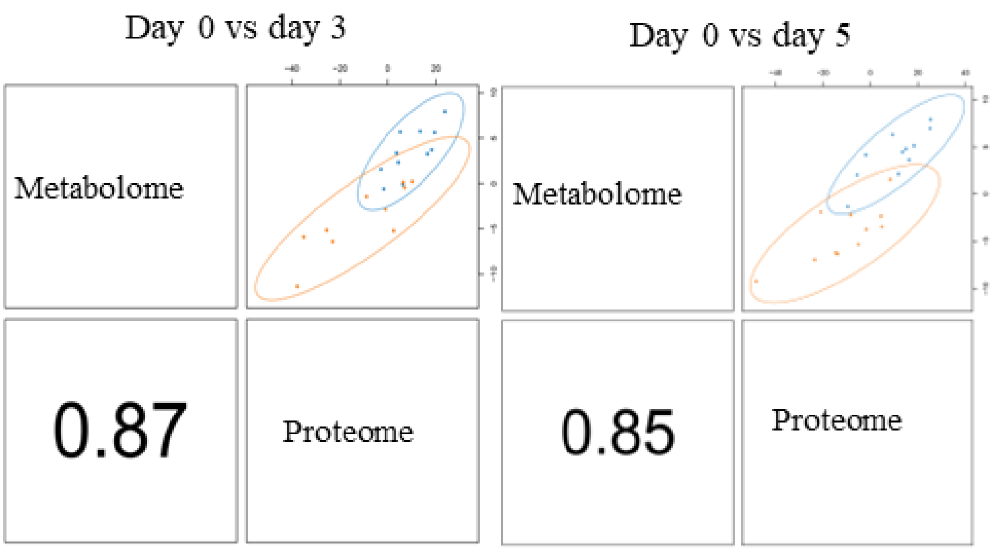

Supplementary figure 1. The consensus component plots via DIABLO algorithm.
